# Experimental and Numerical Study on the Compressive Failure of Composite Laminates with Fiber Waviness Defects

**DOI:** 10.3390/polym13193204

**Published:** 2021-09-22

**Authors:** Yuequan Wang, Shuhua Zhu, Hongshuang Li, Long Zhou, Wentao Yi

**Affiliations:** 1 College of Materials Science and Technology, Nanjing University of Aeronautics and Astronautics, Nanjing 210016, China; wangyuequan@nuaa.edu.cn; 2 College of Aerospace Engineering, Nanjing University of Aeronautics and Astronautics, Nanjing 210016, China; zhoulong@nuaa.edu.cn (L.Z.); ywt188@nuaa.edu.cn (W.Y.)

**Keywords:** composite laminates, fiber waviness, compressive failure load, finite element model, progressive damage, aspect ratio

## Abstract

Fiber waviness defects are found in the inner surface of the hat-shaped stringers manufactured by a process system. In order to establish the acceptance criterion for the stringers with the fiber waviness defects, experimental testing and numerical simulation were carried out in this study. Specially induced fiber waviness defects of four pre-defined severity levels were manufactured and tested. A maximum of a 58.1% drop in compressive failure load is observed for the most severe level in the experimental results. A finite element model with progressive damage method and cohesive zone technique was developed to simulate the failure process and the impact of fiber waviness defects. The numerical simulation results of compressive failure load have a good agreement with experimental results qualitatively and quantitatively. In addition, two simple parameters, i.e., aspect ratio *A/H* and the number of plies with fiber waviness, are proposed to characterize the influence of the fiber waviness on the compressive failure load for the purpose of fast engineering quality checks.

## 1. Introduction

Composite laminates are susceptible to manufacture defects due to their inherent anisotropic characteristics of composite materials and complex manufacturing processes. Fiber waviness defects are the most commonly observed manufacturing defects in the composite laminates, which are introduced unintentionally during the manufacturing process. The presence of fiber waviness defects is well-known to reduce the compressive strength of composite laminates. Therefore, the investigations of fiber waviness defects are important both to evaluate the structural integrity of composite components and to improve manufacturing techniques to reduce the defects.

Many studies have been carried out on the formation of fiber waviness defects and the influence of fiber waviness defects on the mechanical performance of fiber-reinforced composites. Recent studies were summarized in Alves et al. [1] and Kulkarni et al. [2]. The formation of fiber waviness is mainly caused by the selected processing methods and the geometry features of composite structures [3,4,5]. The investigation of the processing parameters by Kugler and Moon showed that the major reason for the formation of fiber waviness in thin laminates was the mismatch of the coefficient of thermal expansion between the tool plate and part [3]. The various sources of in-plane and out-of-plane fiber waviness in the composite structures that occurred at the fabrication stage were detailed by Potter et al. [4]. The tool geometry, such as the inner or external corners, is also a major cause of the out-of-plane fiber waviness [5]. In the filament-winding process, the local buckling of prepreg or wet hoop-wound filament strands is a result of the pressure exerted by the overwrapped layers [6]. Layer waviness occurs in thick cross-ply or multidirectional laminates as a consequence of lamination residual stresses built up during curing [6]. In the molding process of hot drape-forming technology, in-plane slip deformation of fiber layers might be limited in a multilayer prepreg, resulting in in-plane fiber buckling and out-of-plane wrinkling [7]. In the process of automated fiber placement, the laying heads turning on small radii will cause fibers to buckle out-of-plane [8]. The unexpected ply gaps and overlaps due to a contoured mold surface also induce out-of-plane waviness during manual/automated prepreg lamination [9,10]. Based on its direction, fiber waviness can be categorized as in-plane waviness or out-of-plane waviness [11,12]. In-plane waviness, also known as in-plane buckling, involves the cooperative undulation of fibers within the plane of composite laminates [3,13]. Out-of-plane waviness, also known as ply waviness or wrinkling, is identified by the fiber-misalignment through-the-thickness of laminate, resulting in localized wavy regions. Out-of-plane waviness is very commonly observed in thick-section components or curved composite parts [13]. The geometry of out-of-plane waviness is usually characterized with the amplitude, wavelength and waviness angle [14], and simplified with a sine/cosine function or the Gaussian function [15]. The out-of-plane waviness is the focus of the current study because they occur in the hat-shaped composite stringers in an aircraft wing, which is of our interest to design.

Fiber waviness defects were demonstrated by experiments and numerical simulations for reducing the mechanical performance of composite laminates and structures, such as compressive strength, tensile strength, fatigue life. In the open literature, the stiffness and natural frequency of a composite beam had a reduction due to the in-plane fiber waviness [13]. The pristine compressive strength of the laminates also presented a drop due to the out-of-plane fiber waviness defects [16]. It has been reported that the degradation in terms of the tensile stiffness and the failure load of the laminates grew with the increasing height of the out-of-plane fiber waviness [15,17]. The strength of the curved laminates was weakened by the out-of-plane fiber waviness due to the premature failure caused by the local stress concentration around the fiber waviness [18,19]. The ultimate open-hole compressive strength of the CFRP laminates was also reduced by the containing concave waviness defects [20]. Both the in-plane waviness and the out-of-plane waviness reduced the tensile and compressive mechanical properties of composite laminates [21]. Nartey et al. presented a maximum of 21% and 37% drop in the tensile and compressive strength of the composite laminates containing fiber waviness induced by the most severe combination of gaps and overlaps [22]. Wisnom and Atkinson carried out a finite element (FE) analysis of the compressive failure of unidirectional laminates with artificially induced waviness [23]. Garnich and Karami proposed a linear elastic finite element micromechanics model for wavy fiber composite to determine the average stress and strain components [24] and performed a failure study for various types of localized fiber waviness [25]. Lemanski et al. performed the FE analysis on the compressive behavior of the unidirectional composites with a region of misaligned reinforcement [26,27]. Mukhopadhyay et al. built up three-dimensional FE models to predict the tensile, compressive and fatigue failure of quasi-isotropic composite laminates with embedded ply waviness [16,28,29]. Davidson and Waas developed an FE model to study the interaction and influence of defect dimensions on the compressive strength, which allowed the kinking and splitting of composite layers [30]. Ning et al. established an elastic-plastic damage model of composite laminates and simulated the compressive behavior of the composite laminates with out-of-plane waviness subjected to axial compressive loading [31]. Overall, the current investigation of fiber waviness defects shows a trend from unidirectional composite to engineering applications.

In this paper, four configurations of test specimens with different severities of fiber waviness defects were manufactured and tested. The severity of fiber waviness on the specimens was controlled by the customized molds, which were cured together with the specimens. The compression tests were performed following the instructions of ASTM D6641/D6641M-16 [32]. An FE model was developed based on the specimen information. The continuum damage mechanics and cohesive zone models were incorporated in the developed FE model to simulate the failure process caused by waviness defects. Then, the experimental results were used to validate the developed FE model. On this basis, the influence of two proposed waviness parameters on compressive strength is studied by FE simulations.

The purpose of this study is to build up relationships between waviness parameters and compressive failure load drop and provide further insight into the influence of out-of-plane waviness defects on the failure of composite stringers in an aircraft. The organization of the paper is as follows. The manufacturing process of the hat-shaped composite stringer is briefly described in Section 2. Section 3 presents the artificially induced waviness of controlled severity in testing specimens and experimental results. The three-dimensional FE models with the damage formulations are presented in Section 4. A comparison between the testing results and simulated ones is also shown in Section 4. Section 5 discusses the relationship between waviness parameters and compressive failure load drop. Finally, conclusions are drawn based on the above studies in Section 6.

## 2. Manufacture Process of Composite Stringers

**Figure 1:**
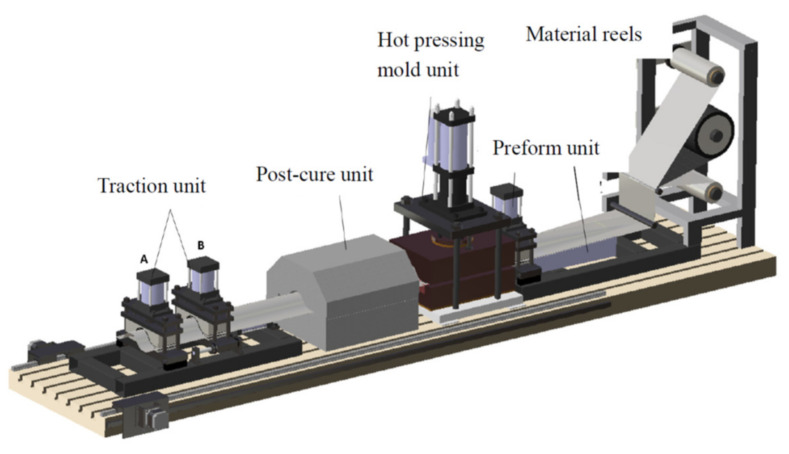
Manufacture process of the stringer.

**Figure 2:**
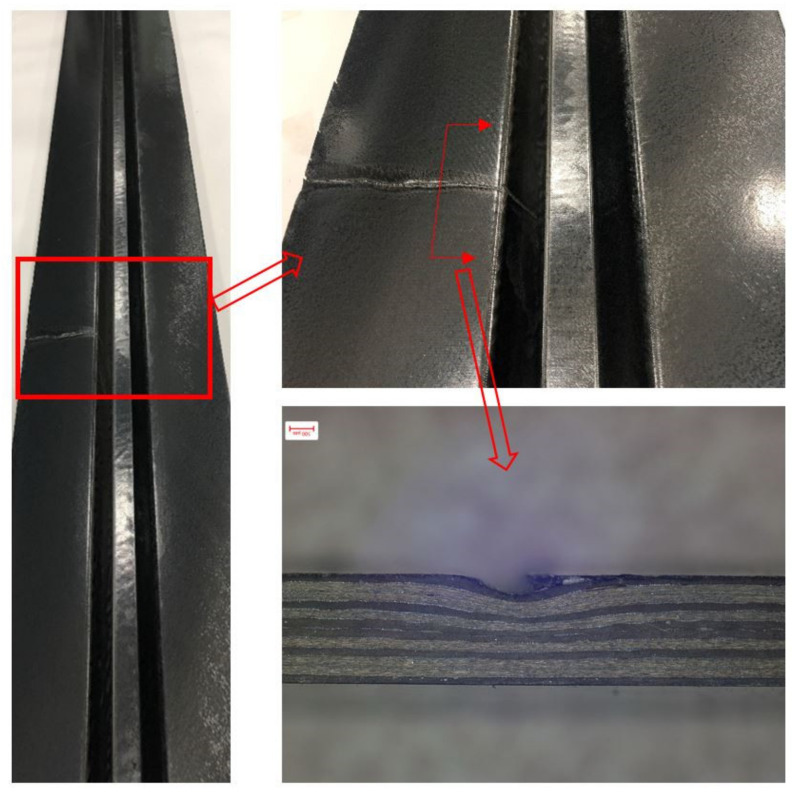
Fiber waviness defects found on the fabricated stringer.

The hat-shaped composite stringers are widely used in the fuselage structures of aircraft. A stepped hot-pressing process (as illustrated in Figure 1) was developed for the purpose of reducing the manufacturing cost of the composite aircraft stringers. The prepreg layers of stringers are fabricated by a customized automatic tape laying machine in advance. The prepreg is then placed between the bottom and top separation films in the unwinding process of material reels. After that, a stringer is formed in the hot-pressing mold unit and moves forward in a designed step and interval with the drag of the traction unit when the hot-pressing mold was opened. However, random fiber waviness defects were observed on the inner surface of the stringer, which were caused by the folding of the separation films. Figure 2 shows a common fiber waviness defect that has a dent shape. It is found that the fiber waviness defects cannot be avoided and are predictable after many improvements to the forming process in the current stage. The quality of manufactured stringers is good except for the presence of fiber waviness defects. Therefore, it is necessary to evaluate the influence of the fiber waviness defect on the mechanical performance of stringers and furtherly provide acceptance criterion for the corresponding stiffened panel design and insight of improving the process to avoid the defects.

## 3. Experimental Study

For the purpose of manufacturing specimens with pre-defined severity waviness defects and specimens without waviness, carefully designed lay-up and forming processes were selected and implemented. Then, mechanical tests were performed to obtain failure modes and loads of specimens with and without fiber waviness.

### 3.1. Manufacturing of Specimens with Waviness

The composite laminates specimens were designed containing four pre-defined severities of fiber waviness defects. For each pre-defined severity, four specimens were produced to reduce the random effects. A group of four specimens without waviness were also manufactured using the same curing process to serve as references against which the waviness-containing specimens were compared for compressive failure load reduction. In total, there were 20 specimens for the experimental study. For the specimens designed to contain fiber waviness, an additional special process was used to introduce the waviness defects so that the severity of the waviness defect can be controlled. To generate the pre-defined severity waviness defects, we designed and machined a group of steel molds. The severity of the waviness can be controlled by varying the aspect ratio of the hills or valleys on the surface of the molds. Prepregs of the required number were laid upon the ply manually, and each ply was pressed to remove any entrapped air and wrinkles. Then, the lay-up was sealed and vacuumed at the edges. Finally, the molds and composite laminates were cured together in an autoclave following the material supplier’s instructions. The stacking sequence is [45/0/−45/90/45/0/−45/0/45/0]_s_. Here, the zero degree is orientated along the direction of the stringer. The composite material used for the specimens is T800/epoxy unidirectional prepregs with a fiber volume ratio of 56.6%. The nominal thickness of each ply is 0.19 mm. Then, the nominal total thickness of the specimens is 3.8 mm. Following the instruction of the standard ASTM D6441/D6641M-16 [32], all the specimens were machined to the length of 140 mm and the width of 12 mm. The unsupported length of the specimens was 12 mm.

**Figure 3:**
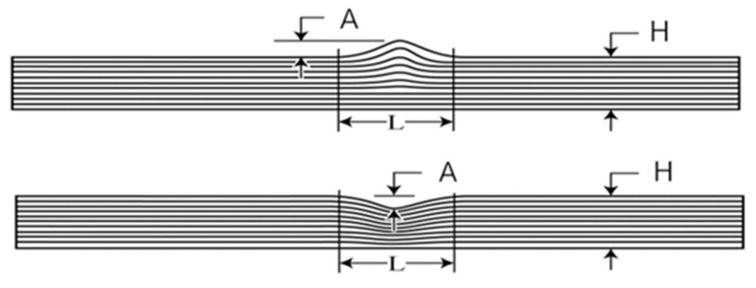
Side-view of specimens containing a fiber waviness defect.

As shown in Figure 3, the nominal thickness of laminates is denoted as *H*. The maximum depth of dent or height of convex is denoted as *A*. In this study, the severity of the fiber waviness defect is characterized by the fiber waviness ratio *A/H*. Four configurations of specimens with different fiber waviness ratios were investigated, which were 10%, 20%, −10% and −20%, respectively. Note that the negative sign represents the concave fold of the fibers; otherwise it is the convex fold of the fibers. The number of defects could be more than one in practice; however, we only introduced one fiber waviness defect for each specimen for our first investigation, either concave fold or convex fold of fibers. The location of defects is in the middle position of a specimen.

### 3.2. Compression Testing

**Figure 4:**
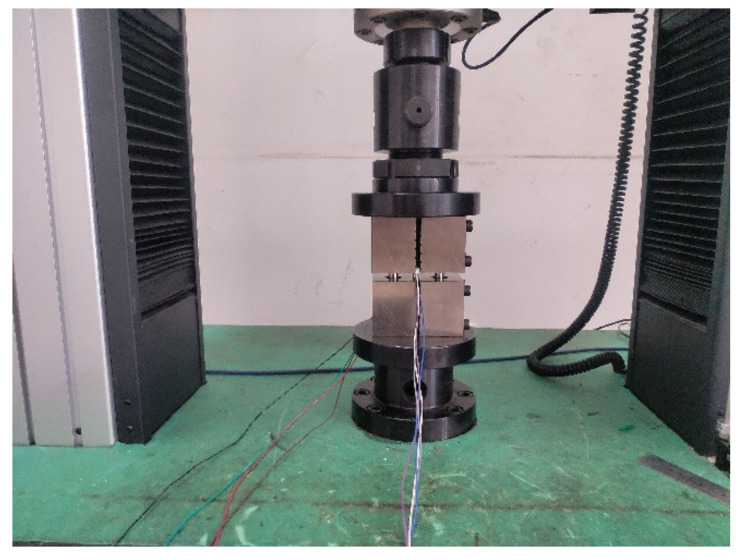
WDWE200D compression test fixture.

The specimens were tested up to failure under compression loading using an electronic universal testing machine (WDWE200D) (Jinan Test Machine Factory, Jinan, China) according to ASTM D6641. The specimens were held by a pair of testing fixtures (Figure 4). It is crucial to install the specimen in the middle of the test machine so that the loading line can be neutral. The specimens were firstly pre-loaded in order to eliminate the installation clearance. Then the specimens were compressed at a constant loading rate of 1 mm/min with the displacement control mode until the final failure occurred. The loading histories were recorded by the computer controller automatically.

### 3.3. Testing Results

**Figure 5:**
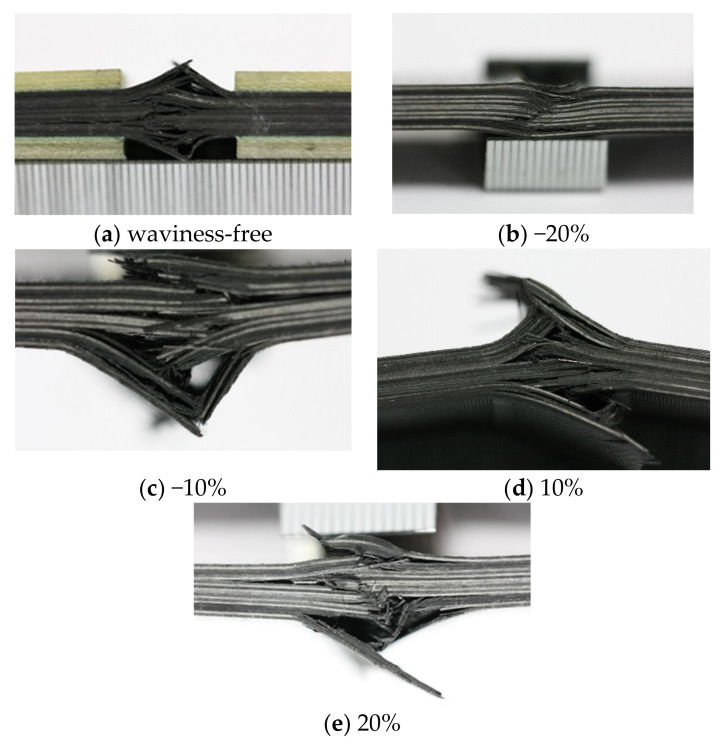
Final state of the failed specimens with different fiber waviness ratios: (**a**) waviness-free, (**b**) −20%, (**c**) −10%, (**d**) 10%, (**e**) 20%.

The failures of all the specimens happened suddenly and were accompanied by an audible noise during the tests. It is as expected that all the specimens failed at the location of the fiber waviness zone. The typical failure modes of all four configurations of specimens are shown in Figure 5. All the fiber waviness defects are placed at the top of the photographs. The bright lines indicate 0° layers in the composite laminates. According to the final state of the failures in Figure 5, the specimens with −20% fiber waviness failed in fiber fracture finally. The −10%, 10% and 20% fiber waviness specimens failed due to delamination and fiber fracture finally. The matrix cracks happened with the fiber fracture failure and delamination failure. Therefore, the fiber fracture is the dominant failure mode for the currently investigated laminates, but the multiple-delamination also accompanied the fiber fractures in most cases. This was further confirmed by the finite element modeling in Section 4 with a wider range of fiber waviness ratios.

**Figure 6:**
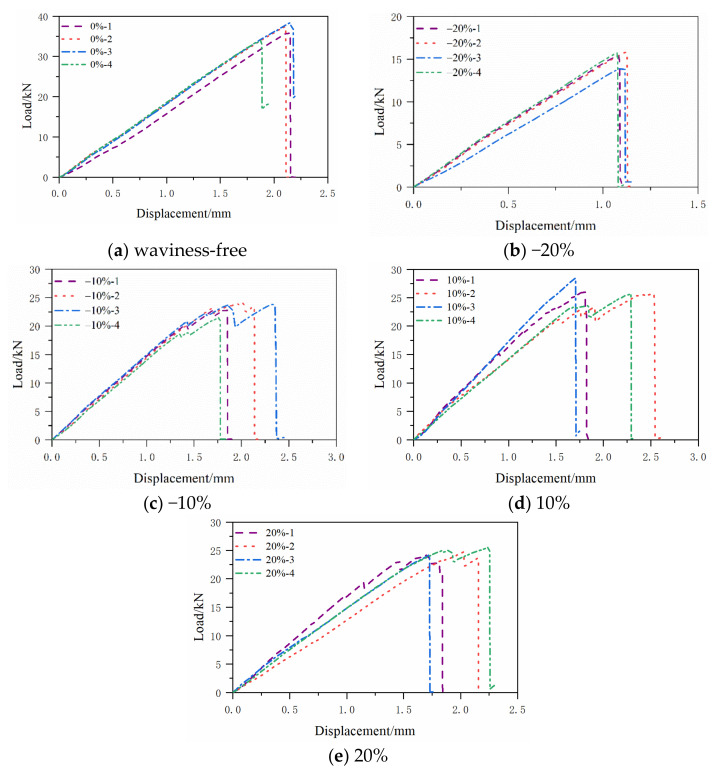
Compressive load-displacement curves with different fiber waviness ratio: (**a**) waviness-free, (**b**) −20%, (**c**) −10%, (**d**) 10%, (**e**) 20%.

The compression load vs. displacement curves obtained from the mechanical tests are given in Figure 6 for the specimens without and with waviness defects, respectively. In the figure legends, the first number represents the severity of the waviness, and the second number represents the numbering of the testing specimen. It can be seen that both specimens show an elastic behavior before the first loading reduction. Furthermore, the load is recovered to a new peak with a new elastic stiffness and is followed by subsequent loading reductions for the cases of −10% and 20% fiber waviness ratios. For the fiber waviness ratio of −20%, the loading reduction only happens once for all four tested specimens, i.e., the load-displacement curves only have one peak followed by a rapid drop in the loading history. While for the fiber waviness ratio of 10%, a mixture of both phenomena is observed.

The compressive failure loads are summarized in Table 1. Compared with waviness-free specimens, a range of significant reductions, about 27–58%, for the final failure load was observed for the specimens containing waviness, which clearly indicated a significant influence of fiber waviness defects on the mechanical capacity of composite laminates as reported in the open literature (for example, Mukhopadhyay et al. reported about a 36% compressive failure load reduction due to an emended wrinkle on quasi-isotropic laminates) [28]. Although all specimens were from the same panel, small variations in the material properties are sufficient to introduce uncertainty in the mechanical tests and are further reflected by the coefficient of variance (CV) of the failure load. The CVs of the mechanical tests are lower than the maximum CV of 6.1% reported in Ref. [28]. This implies the mechanical tests have been performed by a better control scheme considering the statistical meaning.

## 4. Finite Element Modeling

### 4.1. Constitutive Law

#### 4.1.1. Progressive Failure Analysis

**Figure 7:**
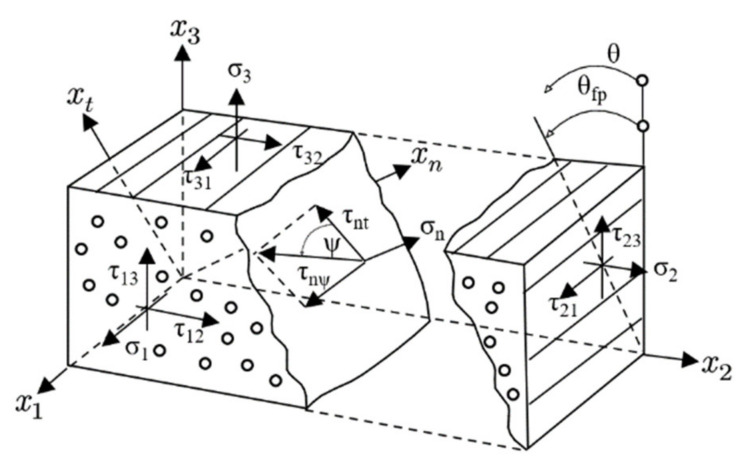
Definition of stresses on the crack plane.

The strength theory of composites takes damage characterization into account, including stress analysis, damage judgment and damage evolution. The continuum damage mechanics (CDM) holds that material damage will directly result in the reduction of carrying capacity. It provides a method that can accurately determine the full range of deterioration in a composite material [33]. To capture the failure process of composites, progressive failure analysis methods have been developed. In this study, the damage in composites was predicted with Puck’s theory [34,35], which is one of the most popular criteria used to predict damage and distinguish the failure modes. Here, Puck’s theory was coded as VUMAT subroutines in ABAQUS/Explicit, which assumes that the brittle fracture of the matrix in unidirectional composite laminates will generate a crack plane parallel with the fiber direction (Figure 7). The coordinate system 1-2-3 is the principal axes system of each lamina, while the l-n-t is the matrix damage crack plane system. Therefore, the relationship between stress components in two coordinates systems can be written as follows:

(1) $$\begin{array}{l} \sigma_{n}\left(\theta \right)=\sigma_{2}\cos^{2}\theta +\sigma_{3}\sin^{2}\theta +2\tau_{23}\sin\theta \cos\theta \\ \tau_{nt}\left(\theta \right)=\left(\sigma_{3}-\sigma_{2}\right)\sin\theta \cos\theta +\tau_{23}\left(\cos^{2}\theta -\sin^{2}\theta \right)\\ \tau_{nl}\left(\theta \right)=\tau_{13}\sin\theta +\tau_{12}\cos\theta \end{array}$$

where *θ* is the so-called fracture angle.

In CDM, the damage variable based on the residual modulus of elasticity is defined as:

(2) $$d=\left(E^{0}-E^{d}\right)/E^{0}$$

where $E^{d}$ is the effective modulus of elasticity of damaged configuration, $E^{0}$ is the effective modulus of elasticity of fictitious undamaged configuration.

In Puck’s theory [35], the material fracture is represented by the stress exposure $f_{E}$. If $f_{E}<1$, there is no damage, otherwise if $f_{E}\geq 1$, the material has damage occurred.

Puck considered that fiber failure (FF) and inter-fiber failure (IFF) must be treated by different criteria. According to the IFF hypothesis, two different fracture conditions for IFF are required.

(3) $$\begin{array}{c} f_{E_{IFF}}(\theta )=\{\begin{array}{c} {\left[{\left(\frac{1}{R_{⊥}^{t}}-\frac{p_{⊥\psi }^{t}}{R_{⊥\psi }^{A}}\right)}^{2}\sigma_{n}^{2}(\theta )+{\left(\frac{\tau_{nt}(\theta )}{R_{⊥⊥}^{A}}\right)}^{2}+{\left(\frac{\tau_{nl}(\theta )}{R_{⊥\|}}\right)}^{2}\right]}^{\frac{1}{2}}+\frac{p_{⊥\psi }^{t}}{R_{⊥\psi }^{A}}\sigma_{n}(\theta ),\left(\sigma_{n}(\theta )\geq 0\right)\\ {\left[{\left(\frac{p_{⊥\psi }^{c}}{R_{⊥\psi }^{A}}\sigma_{n}(\theta )\right)}^{2}+{\left(\frac{\tau_{nt}(\theta )}{R_{⊥⊥}^{A}}\right)}^{2}+{\left(\frac{\tau_{nl}(\theta )}{R_{⊥\|}}\right)}^{2}\right]}^{\frac{1}{2}}+\frac{p_{⊥\psi }^{c}}{R_{⊥\psi }^{A}}\sigma_{n}(\theta ),\left(\sigma_{n}(\theta )<0\right) \end{array} \end{array}$$

where $\frac{p_{⊥\psi }^{t,c}}{R_{⊥\psi }^{A}}=\frac{p_{⊥⊥}^{t,c}}{R_{⊥⊥}^{A}}\cos^{2}\psi +\frac{p_{⊥\|}^{t,c}}{R_{⊥\|}^{A}}\sin^{2}\psi$, $R_{⊥⊥}^{A}=\frac{R_{⊥}^{c}}{2\left(1+p_{⊥⊥}^{c}\right)}$, $\cos^{2}\psi =\frac{\tau_{nt}^{2}}{\tau_{nt}^{2}+\tau_{nl}^{2}}$, $\sin^{2}\psi =\frac{\tau_{nl}^{2}}{\tau_{nt}^{2}+\tau_{nl}^{2}}$.

For fiber fracture (FF), the maximum stress failure conditions are given as:

(4) $$f_{E_{FF}}=\left\{\begin{array}{l} \frac{\sigma_{1}}{R_{\|}^{t}}\;\left(\sigma_{1}\geq 0\right)\\ -\frac{\sigma_{1}}{R_{\|}^{c}}\;\left(\sigma_{1}<0\right) \end{array}\right.$$

In Equations (3) and (4), ‘‖,⊥’ indicate parallel to fiber and transverse (perpendicular) to fiber directions. The two shear stresses $\tau_{nt}$ and $\tau_{nl}$ can be merged to one shear stress $\tau_{n\psi }$, characterized by the angle ψ. $R^{A}$ indicates fracture resistance. $R_{⊥}^{t}$ and $R_{⊥}^{c}$ indicate the tensile and compressive strength of unidirectional lamina transverse to the fiber direction. $R_{⊥\|}$ represents the in-plane shear strength of the lamina. $p_{⊥\|}^{t,c}$ indicates the inclination of ($\sigma_{2}$,$\tau_{21}$)-fracture curve at $\sigma_{2}$ = 0 (*t* for the range $\sigma_{2}$> 0 and *c* for the range $\sigma_{2}$ < 0). $p_{⊥⊥}^{t,c}$ indicates the inclination of ($\tau_{nt}$,$\sigma_{n}$)-fracture curve at $\sigma_{2}$ = 0 (*t* for the range $\sigma_{2}$> 0 and *c* for the range $\sigma_{2}$ < 0).

**Figure 8:**
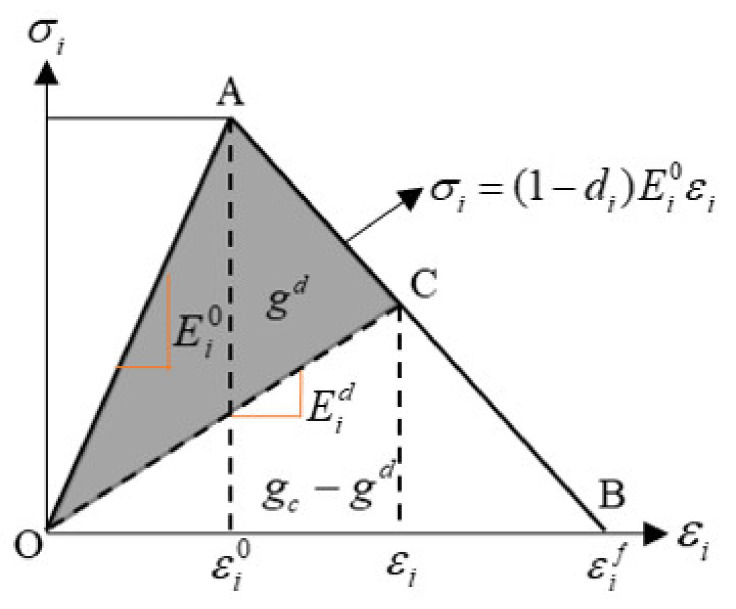
The process of material damage evolution.

Damage evolution, based on energy dissipation during the damage process, assumes that damage is characterized by progressive degradation of material stiffness, leading to material failure. The bilinear softening law was employed in this paper (Figure 8), where $g_{c}$ is the area of the entire triangle, and $g^{d}$ is the shaded area.

The positive slope of the stress-strain curve prior to damage initiation (point A) corresponds to linear elastic material behavior, where the modulus of elasticity and strain are $E_{i}^{0}$ and $\varepsilon_{i}^{0}$. The negative slope after damage initiation is achieved by the evolution of the respective damage variable $d_{i}$ according to the below formula:

(5) $$d_{i}=\max\left\{0,\min\left\{1,\frac{\varepsilon_{i}^{f}\left(\varepsilon_{i}-\varepsilon_{i}^{0}\right)}{\varepsilon_{i}\left(\varepsilon_{i}^{f}-\varepsilon_{i}^{0}\right)}\right\}\right\}$$

where $\varepsilon_{i}^{0}$ and $\varepsilon_{i}^{f}$ are the strains corresponding to the damage initiation and the complete failure. The damage variable $d_{i}$ ranges from 0–1. $d_{i}=0$ corresponds to the undamaged. $d_{i}=1$ corresponds to the complete failure. The material properties of the T800/epoxy composite used in this study are given in Table 2.

#### 4.1.2. Cohesive Interfaces for Delamination

The interfaces were modeled using a 3D cohesive interface constitutive in the traction-separation law embedded in Abaqus/Explicit. The opening (mode I) and sliding (mode II) displacements between the top and bottom surfaces of the cohesive element were related to the corresponding stress components by high interfacial stiffness value $K_{I}$ and $K_{\mathrm{II}}$. The material parameters of the cohesive zones are given in Table 3. The cohesive interfacial stiffness and strength were estimated because they cannot be measured directly. The quadratic stress criterion was used to detect damage initiation.

(6) $${\left(\frac{\langle \sigma_{nn}\rangle }{N_{\max}}\right)}^{2}+{\left(\frac{\sigma_{ss}}{S_{\max}}\right)}^{2}+{\left(\frac{\sigma_{tt}}{T_{\max}}\right)}^{2}=1$$

where $\sigma_{nn}$ is the normal component of stress in the cohesive layer, $\sigma_{ss}$ and $\sigma_{tt}$ are two shear traction components of stress in cohesive layer, while $N_{\max}$,$\;S_{\max}$ and $T_{\max}$ are the mode I, mode II and mode III failure initiation stresses. Here, the Macaulay bracket on $\sigma_{nn}$ indicates that negative normal traction component does not contribute to traction-separation of the cohesive layer. After damage initiation is detected at the interface, the bilinear damage evolution law is used to degrade the stiffness of the cohesive elements. The damage variable *d*_delam_ was defined by:

(7) $$d_{\mathrm{delam}\;}=\max\left\{0,\min\left\{1,\frac{\delta_{m}-\delta_{m}^{i}}{\delta_{m}^{f}-\delta_{m}^{i}}\right\}\right\}$$

where $\delta_{m}$ is the effective interfacial mixed-mode displacement. The superscript ‘*i*’ and ‘*f*’ indicate their values corresponding to damage initiation and complete failure, respectively. The damage variable, *d*_delam_, was used to degrade the interfacial stiffness linearly from 0 (no damage) to 1 (complete failure) following a mixed-mode delamination. After damage initiation, a power-law fracture energy criterion was used for the damage evolution.

(8) $${\left(\frac{G_{I}}{G_{\mathrm{IC}}}\right)}^{\alpha }+{\left(\frac{G_{\mathrm{II}}}{G_{\mathrm{IIC}}}\right)}^{\alpha }=1$$

where $G_{I}$ and $G_{\mathrm{II}}$ are the strain energy release rates for pure mode I and pure mode II, respectively. $G_{\mathrm{IC}}$ and $G_{\mathrm{IIC}}$ are the critical strain energy release rates for mode I and mode II, respectively, and α is the power.

### 4.2. Meshing and Boundary Conditions

**Figure 9:**
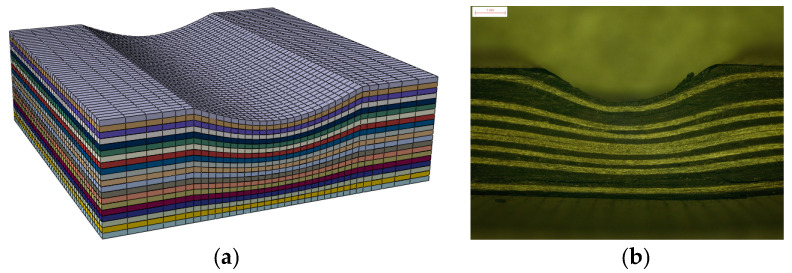
FE mesh and side view of specimen with −20% fiber waviness specimen. (**a**) FE mesh of specimen (**b**) side view of the test specimen.

The composite plies were modeled using 3D 8-noded solid reduced-integration elements (C3D8R). There were one-layer elements for each ply in the through-the-thickness direction. The ‘enhanced’ hourglass control option was used to avoid the appearance of spurious zero-energy hourglass modes in the reduced integration C3D8R elements. A layer of zero-thickness 8-noded cohesive COH3D8 elements was inserted between two layers of composite elements. A finer mesh, 0.25 × 0.25 mm^2^ in-plane dimension, was employed at the zone containing fiber waviness. In addition, a relatively coarser grid (the largest size scale reached 0.75 × 0.25 mm^2^ in-plane dimension) was used in the other zones to improve the efficiency of the calculation. To improve the computing efficiency, only the gauge length of 12 mm of the specimens was constructed. Totally, a typical FE model of specimen consisted of 34,560 C3D8R elements and 32,832 COH3D8 elements. The mesh of a typical specimen and the actual side view of the specimen are presented in Figure 9. This mesh combination has passed a mesh sensitivity checking, which can generate mesh-independent responses.

**Figure 10:**
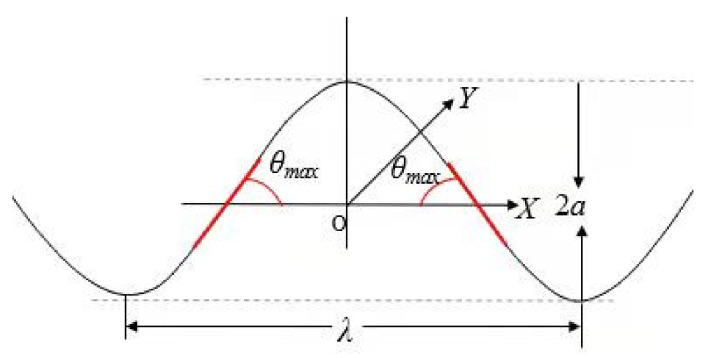
Schematic diagram of fiber waviness.

The FE models for different severities were built in Abaqus/Explicit and implemented with quasi-static analysis and the explicit dynamic solver. The calculated kinematic energy was less than 5% of the internal energy of the model, which indicated that the quasi-static simulation was credible. In this study, the geometrical profile of fiber waviness is represented as the cosine function along the through-thickness direction with a wavelength of λ and an amplitude of 2*a*, as shown in Figure 10.

**Figure 11:**
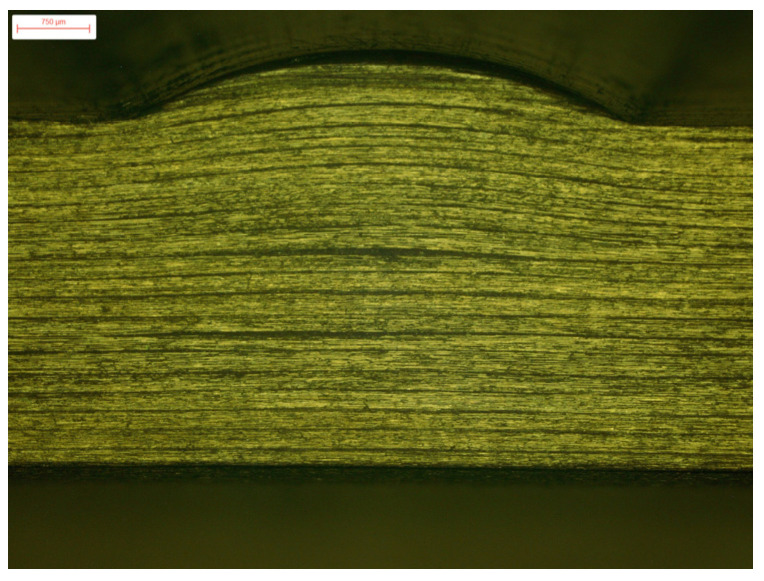
Scanning of the convex fiber waviness.

The scanning of the convex fiber waviness shows that the fibers of the lower half composite laminate had no waviness along the through-thickness direction of the specimens, while the upper-half fibers had waviness along the through-thickness direction, as shown in Figure 11.

The nodal coordinates of the convex fiber waviness model in the direction of through-thickness are defined as:

(9) $$Z_{W}=Z_{0}+\Delta Z$$

where $Z_{W}$ is the nodal coordinate of the through-thickness direction of the fiber waviness model, $Z_{0}$ is the nodal coordinate of the through-thickness direction of the waviness-free model and ΔZ is expressed as:

(10) $$\Delta Z=\left\{\begin{array}{l} \left(\mathrm{sgn}\left(z-\frac{H}{2}\right)+1\right)\frac{BT}{2}\frac{A}{H}\cos\left(\frac{2\pi }{\lambda }\right)\;\;\;if-\frac{\lambda }{4}\leq x\leq \frac{\lambda }{4}\\ 0\;\;\;\;\;\;\;\;\;\;\;\;\;\;\;\;\;\;\;\;\;\;\;\;\;\;\;\;\;\;\;\;\;\;\;\;\;\;\;\;\;\;\;\;\;\;\;\;\;\;\;otherelse \end{array}\right.\;$$

Note that sgn(x) is the symbolic function:

(11) $$\mathrm{sgn}=\left\{\begin{array}{l} 1\;\;\;\;\;\;\;\;\;\;\;\;\;\;\;\;\;\;\;\;\;\;\;\;\;\;\;\;x>0\\ 0\;\;\;\;\;\;\;\;\;\;\;\;\;\;\;\;\;\;\;\;\;\;\;\;\;\;\;\;x=0\;\\ -1\;\;\;\;\;\;\;\;\;\;\;\;\;\;\;\;\;\;\;\;\;\;\;\;\;x<0 \end{array}\right.$$

where x is the coordinate of the model length direction. λ is the wavelength of the waviness, and the wavelength of the fiber waviness on the specimens is half the wavelength. *A* is the convex height of the fiber waviness in the direction of through-thickness, and *H* is the theoretical thickness of the model. *B* is the amplitude in the direction of through-thickness, which is one on the top surface of the FE model, linearly decays in the direction of through-thickness, and takes to zero at the middle-plane position of the composite laminates. *T* is equivalent to *H*, which is also the thickness of the model. *A/H* is used here to define the severity of fiber waviness.

For the geometrical profile of the concave fiber waviness, it is roughly the same as that of the convex fiber waviness model. The curvature of the concave fiber waviness gradually decays from the top surface to its subsequent plies. Therefore, the severity of the concave fiber waviness on the top layer was the largest, and the bottom layer did not have any fiber waviness. The nodal coordinate of the concave fiber waviness model in the through-thickness direction is defined as follows:

(12) $$Z_{W}=Z_{0}-\Delta Z$$

where $Z_{W}$ is the nodal coordinate along the through-thickness direction of the fiber waviness model, $Z_{0}$ is the nodal coordinate along the through-thickness direction of the FE model without waviness defect and ΔZ is expressed as:

(13) $$\Delta Z=\left\{\begin{array}{l} \frac{BT}{2}\frac{A}{H}\cos\left(\frac{2\pi }{\lambda }\right)\;\;\;\;\;\;\;\;\;\;\;\;\;\;\;\;\;\;if-\frac{\lambda }{4}\leq x\leq \frac{\lambda }{4}\\ 0\;\;\;\;\;\;\;\;\;\;\;\;\;\;\;\;\;\;\;\;\;\;\;\;\;\;\;\;\;\;\;\;\;\;\;\;\;\;otherelse \end{array}\right.\;$$

Note that the meanings of symbols in Equation (13) are identical to those in the convex fiber waviness model. In the concave fiber waviness model, parameter *B* is one at the top layer and zero at the bottom layer.

### 4.3. FE Analysis Results

The failure loads of the specimens predicted by finite element analysis are summarized in Table 4. It can be seen that the simulation values match those of the experimental data very well, and the relative errors are all within 8%. Furthermore, the developed models predicted slightly lower failure loads for all five cases, which is conservative for engineering design.

**Figure 12:**
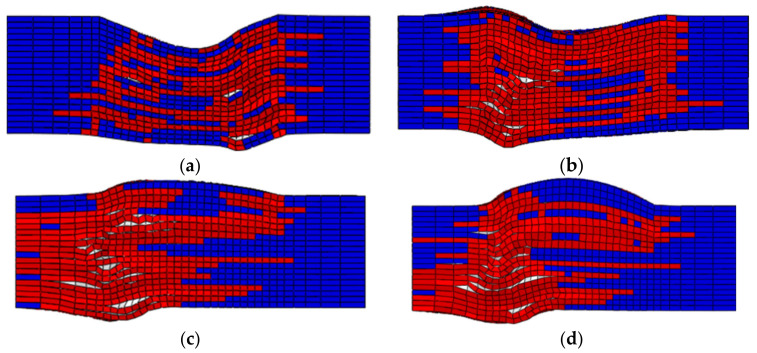
Simulation failure modes with different *A*/*H*: (**a**) −20%, (**b**) −10%, (**c**) 10%, (**d**) 20%.

**Figure 13:**
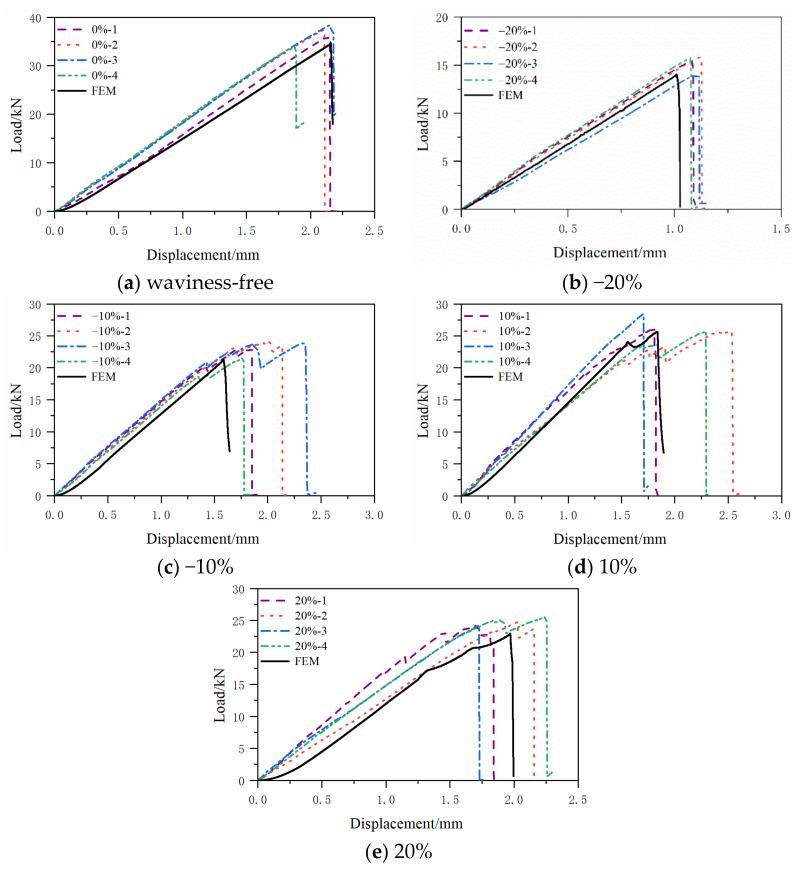
Comparison of compressive load-displacement curves of experiments and simulations. (**a**) waviness-free, (**b**) −20%, (**c**) −10%, (**d**) 10%, (**e**) 20%.

Figure 12 shows the final failure status predicted by the numerical simulation on the four investigated specimens with fiber waviness defects. The elements in blue are the undamaged regions. The elements in red are the failure regions. The elements in white are the delamination regions. All the fiber waviness defects are at the top of these figures. Comparing Figure 5 and Figure 12, it can be found that the failure modes of the convex and concave fiber waviness model obtained by FE simulation are similar to the failure situation of the experiment, i.e., the areas of fiber fracture and delamination on the flat side (bottom of each figure) are larger than the side with fiber waviness (top of each figure). The severity levels of 20%, 10% and −10% have more regions of the fiber fracture and delamination along the through-thickness direction than that in the severity level of −20%. The severity level of −20% has a premature failure of the fiber due to the bending around the minimum thickness location, which causes the lowest level of the failure load comparing to the severity levels of 20%, 10% and −10%. In addition, most of the damage occurs around the origin of the misalignment angle, which has been widely used to define the severity of convex waviness defects in the open literature. Figure 13 presents the compressive load-displacement curves for the five severity levels of waviness. For the waviness-free specimen, the FE result and test results show only one peak load (Figure 13a). It means that the fibers in the layers failed almost at the same moment under the compression loading condition. A sharp load dropping is also found in the load-displacement curve of the −20% severity case (Figure 13b). As stated in the previous section, the fiber failure of the −20% severity specimen is caused by the bending of the specimen. No load redistribution occurred before the final failure of the specimen. The FE model also predicted one peak load along the whole loading phase. For the severity of −10%, the predicted load-displacement curve has one load peak, while multiple load peaks are observed on the testing curves (Figure 13c). The dominant failure mode of the −10% severity specimen predicted by FE analysis (Figure 12b) is still dominated by fiber fracture caused by bending around the concave fiber waviness location. According to the test result (Figure 5c), there are several load redistributions processed before the final failure. That means there is a transit of the dominant failure mode from the severity of −20% to −10%. The developed FE model may not capture the load redistribution of the specimen with the severity −10% waviness well. However, for the other two severity levels of 10% and 20%, all simulation results show multiple load peaks, which reveals there are several load redistribution processes involved in the progressive, cumulative damage analysis. This agrees with the experimental results shown in Figure 13d,e. These figures proved that the FE model in this paper can predict the compressive failure load and the failure mode well by the comparison of the load-displacement curves and the failure mode from the investigated experiments.

It can be observed that the predicted curves of FE simulation and their corresponding tests have a similar gradient and displacement before the moment of failure. In other words, similar global stiffness has been obtained by the experiments and FE simulation.

## 5. Parametric Study

### 5.1. Influence of A/H

A parametric study was carried out to investigate the influence of *A/H* on the compression failure load of this type of laminates for the aircraft stringers. Another purpose of this parametric study was to set up an acceptance criterion for a fast check on the quality of a manufactured stringer. In the previous sections, we have validated that the developed numerical models can predict the compressive failure load and failure mode well. In order to study the effect of *A/H* on the compressive failure load, 24 FE models were established with similar mesh settings, in which *A/H* ranged from −35.0% to 35.0%, with a step size of 2.5%. The computational results are listed in Table 5.

**Figure 14:**
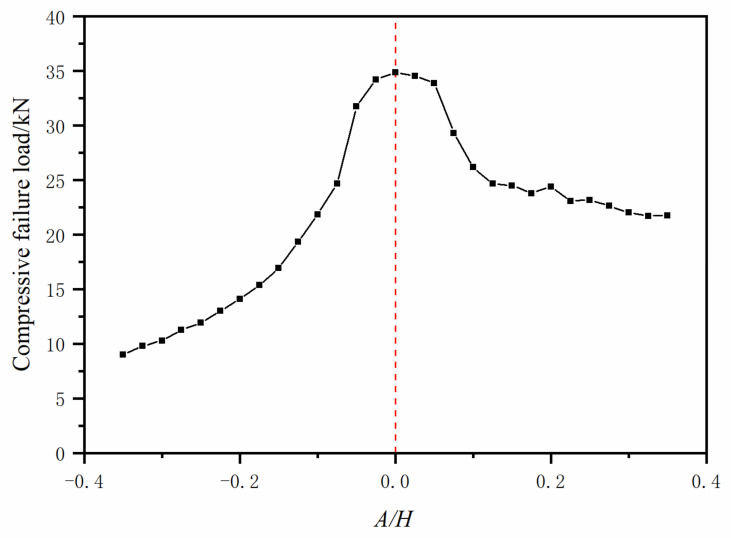
Compressive failure loads vs. aspect ratios.

It can be seen from Table 5 and Figure 14 that whether the fiber waviness mode is convex or concave, the increase of the absolute value of *A/H* will lead to the decrease of compressive failure load. The dropping trend is similar to a bell-shaped curve with the origin where there is no waviness. However, the concave waviness defect has a larger influence than the convex waviness defect (Figure 14). In addition, for the range from −5% to 5% in severity levels, the fiber waviness has little effect on the compressive failure load, and the predicted compressive failure load only decreases by about 3% when compared with the results from the experiment and FE model without a waviness defect. As a conclusion, we selected the 5% severity level as the acceptance criterion during the aircraft stringers’ manufacture. For the convex waviness, between 5% and 12.5% severity levels, there is a fast decline in the compressive failure load. When the aspect ratio *A/H* is larger than 12.5%, the curve is becoming relatively flat, but it still shows a downward-sloping trend. For the concave waviness, a rapid reduction trend is observed from −5.0% to about −20%, and then, the slope of the curve becomes small.

### 5.2. Influence of the Number of Plies with Fiber Waviness

For the convex fiber waviness, only the upper half plies were assumed to contain out-of-plane waviness in the developed FE model in Section 4.2. This means that the upper 10 plies of the laminate were modeled to contain waviness defects in the previous sections. Therefore, a parametric study was carried out to determine the influence of *n*, i.e., the number of plies with fiber waviness, on the compressive failure load. Here *n* is viewed as an additional parameter for the convex waviness case.

**Figure 15:**
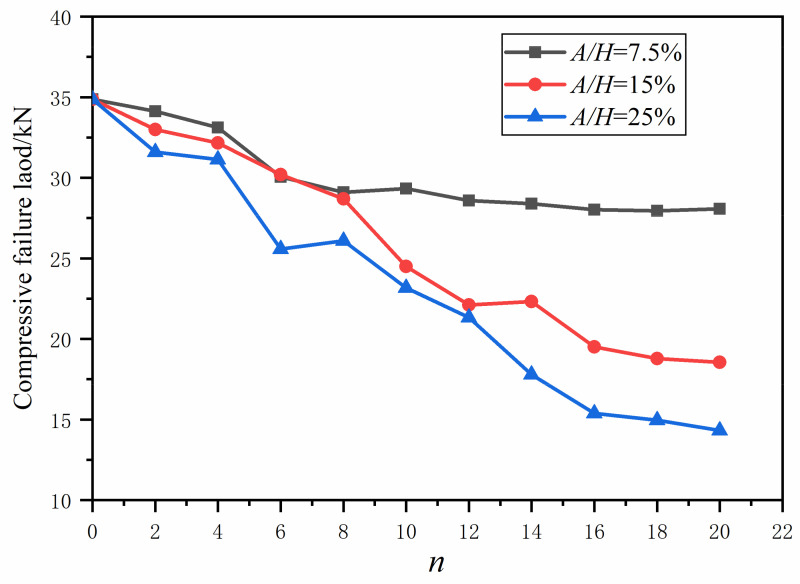
Compressive failure loads vs. *n.*

For this purpose, the FE models were developed with the same overall dimensional setting, but with *n* varying from 2 to 20 with a step size of 2. Here the fiber waviness ratio was fixed at 7.5%, 15.0% and 25.0%, which are considered to represent low, moderate and large values for the severity level. The computational results for the compressive failure load are reported in Table 6 and Figure 15.

For *A*/*H* = 7.5%, the growth of *n* induced a slight reduction in the compressive failure load as shown in Figure 15. In addition, the percentage difference is within 3%, and the compressive failure load is almost unchanged when *n* is larger than eight.

For *A*/*H* = 15.0%, it is clear that the increase of *n* leads to the decrease of the compressive failure load of the composite laminate with waviness defect. At the beginning stage (*n* = 2, 4, 6 and 8), the load vs. *n* curve is going down slightly. When *n* changes from 8 to 12, there is a fast decline in the compressive failure load. Furthermore, the compressive failure load has two step shapes for *n* taking 12–14, 16–20, and there is a small drop in the compressive failure load between them.

For *A*/*H* = 25.0%, the growth of *n* with a large value of the aspect ratio *A/H* resulted in a fast dropping of the compressive failure load, which presents a similar trend to when *A/H* is 15% (Figure 15).

The above analysis indicates that the number of plies containing fiber waviness has a minor influence on the compressive failure load when the aspect ratio is small, while it can lead to a serious impact on the quality of the composite laminates when the aspect ratio is moderate or large. This discovery is also helpful for the building of the acceptance criterion for a fast quality check on the composite stringers in engineering applications.

## 6. Conclusions

Aiming at the hat-shaped composite stringers in a civil aircraft, experimental testing and numerical simulations have been carried out to investigate the influence of out-of-plane waviness defects, which are commonly observed during their manufacturing process. The waviness-free laminated specimen and the specimens with pre-defined waviness defects have been carefully produced with the designed lay-up as in the composite stringers. Mechanical tests show that the specimens with waviness defects exhibited two principal failure modes in the wrinkle region, which may result in a significant reduction in the compressive capacity of the stringers. Additionally, the inter-ply failure mode was observed for all severity cases, while the fiber fracture failure mode seems to dominate the worst severity case (i.e., *A*/*H* = −20%).

A dedicated FE model was developed with a special element mesh methodology to accommodate the waviness profiles. A significant drop of about 37–58% of the compressive failure load was observed for the specimens with waviness defects in both the numerical simulation and experimental results. The FE model was also used to predict the compressive failure load for a large range of severity levels, which generated relationships between the compressive failure load and the waviness parameters.

Several conclusions can be drawn from the current study:

(1) Experiments and FE simulations were carried out with the fiber waviness ratio *A/H* of −20%, −10%, 10%, 20% and waviness-free specimens. The numerical results and failure modes have a good agreement with those from testing. Therefore, the 3D FE model in this paper can be used to predict the compressive failure load and failure mode of composite laminates with out-of-plane waviness defects.
(2) With the increase of the fiber waviness ratio *A/H* (absolute value), the compressive failure load of both convex and concave fiber waviness decreases. It also shows a nonlinear relationship between them. For the current lay-up, the fiber waviness ratio *A/H* has little influence on the compressive failure load when it is less than 5%. Therefore, this value may be identified as an acceptance criterion during the manufacturing process of the composite stringers, beyond which a significant reduction of compressive failure load may occur.
(3) It seems that the effect of the number of plies with fiber waviness *n* varies under different *A/H* conditions for convex fiber waviness. We investigated a low, moderate and high level of *A/H* (i.e., A/H = 7.5%, 15.0% and 25.0%). When it is 7.5%, the compressive failure load only has a slight reduction as *n* increases. In addition, the curve tends to be flat when *n* >= 6.

Further work will involve applying the developed FE model for the prediction of the failure mechanism and mode of the aircraft stringers with random fiber waviness defects from a statistical perspective.

## Figures and Tables

**Table 1:** Compression test results.

Fiber Waviness Ratio	Failure Load		Failure Load Decreasing (%)
	Mean (kN)	CV (%)	
0% (waviness-free)	36.53	5.1	0
−20%	15.27	5.3	58.1
−10%	23.21	5.1	36.5
10%	26.55	4.9	27.1
20%	24.73	2.6	32.3

**Table 2:** Material properties of the T800/epoxy composite.

$E_{1}(\mathrm{MPa})$	$E_{2}=E_{3}(\mathrm{MPa})$	$G_{12}\;$ $=G_{13}(\mathrm{MPa})$	$G_{23}(\mathrm{MPa})$	$\nu_{12}$ $=\nu_{13}$ $=\nu_{23}$
142500	8540	4340	3235	0.32
$X_{t}$(MPa)	$X_{c}$(MPa)	$Y_{t}$(MPa)	$Y_{c}$(MPa)	S(MPa)
2737	1585	86.4	212.8	110

**Table 3:** Material parameters of the cohesive elements.

$K_{I}(N/{\mathrm{mm}}^{3})$	$K_{\mathrm{II}}(N/{\mathrm{mm}}^{3})$	$N_{\max}(\mathrm{MPa})$	$S_{\max}(\mathrm{MPa})$	$T_{\max}(\mathrm{MPa})$
5×10^5^	3×10^5^	50	90	90
$G_{\mathrm{IC}}\left(N/\mathrm{mm}\right)$	$G_{\mathrm{IIC}}\left(N/\mathrm{mm}\right)$	α		
0.3	0.9	1		

**Table 4:** Experimental and simulation results of fiber waviness specimens.

*A/H*	Simulation Value/kN	Experimental Value/kN	Error
0%	34.86	36.53	−4.57%
−20%	14.08	15.27	−7.79%
−10%	21.85	23.21	−5.86%
10%	26.16	26.55	−1.47%
20%	24.39	24.73	−1.37%

**Table 5:** Predicted compressive failure load with different *A/H.*

*A/H*	Compressive Failure Load/kN	*A/H*	Compressive Failure Load/kN
35.0%	21.76	−35.0%	9.01
32.5%	21.72	−32.5%	9.79
30.0%	22.01	−30.0%	10.28
27.5%	22.64	−27.5%	11.29
25.0%	23.17	−25.0%	11.92
22.5%	23.07	−22.5%	13.01
20.0%	24.39	−20.0%	14.08
17.5%	23.79	−17.5%	15.37
15.0%	24.49	−15.0%	16.92
12.5%	24.66	−12.5%	19.35
10.0%	26.16	−10.0%	21.85
7.5%	29.32	−7.5%	24.67
5.0%	33.89	−5.0%	31.73
2.5%	34.55	−2.5%	34.21

**Table 6:** Compressive failure load (kN) with different *n* for positive aspect ratios.

n	*A*/*H* = 7.5%	*A*/*H* = 15%	*A*/*H*= 25%
2	34.12	32.99	31.60
4	33.11	32.16	31.13
6	30.06	30.20	25.58
8	29.10	28.69	26.09
10	29.32	24.49	23.17
12	28.59	22.10	21.33
14	28.39	22.32	17.78
16	28.01	19.51	15.38
18	27.95	18.78	14.96
20	28.07	18.55	14.32

## Data Availability

The data presented in this study are available on reasonable request from the corresponding author. The data are not publicly available due to the restrictions of the funding.
